# Automatic depression diagnosis through hybrid EEG and near-infrared spectroscopy features using support vector machine

**DOI:** 10.3389/fnins.2023.1205931

**Published:** 2023-08-24

**Authors:** Li Yi, Guojun Xie, Zhihao Li, Xiaoling Li, Yizheng Zhang, Kai Wu, Guangjian Shao, Biliang Lv, Huan Jing, Chunguo Zhang, Wenting Liang, Jinyan Sun, Zhifeng Hao, Jiaquan Liang

**Affiliations:** ^1^School of Mechatronic Engineering and Automation, Foshan University, Foshan, China; ^2^Department of Psychiatry, The Third People’s Hospital of Foshan, Foshan, China; ^3^Department of Psychiatry, The Third Affiliated Hospital of Foshan University, Foshan, China; ^4^School of Medicine, Foshan University, Foshan, China; ^5^School of Biomedical Sciences and Engineering, South China University of Technology, Guangzhou, China; ^6^College of Science, Shantou University, Shantou, China

**Keywords:** depression, EEG, brain network, functional near-infrared spectroscopy, machine learning

## Abstract

Depression is a common mental disorder that seriously affects patients’ social function and daily life. Its accurate diagnosis remains a big challenge in depression treatment. In this study, we used electroencephalography (EEG) and functional near-infrared spectroscopy (fNIRS) and measured the whole brain EEG signals and forehead hemodynamic signals from 25 depression patients and 30 healthy subjects during the resting state. On one hand, we explored the EEG brain functional network properties, and found that the clustering coefficient and local efficiency of the delta and theta bands in patients were significantly higher than those in normal subjects. On the other hand, we extracted brain network properties, asymmetry, and brain oxygen entropy as alternative features, used a data-driven automated method to select features, and established a support vector machine model for automatic depression classification. The results showed the classification accuracy was 81.8% when using EEG features alone and increased to 92.7% when using hybrid EEG and fNIRS features. The brain network local efficiency in the delta band, hemispheric asymmetry in the theta band and brain oxygen sample entropy features differed significantly between the two groups (*p* < 0.05) and showed high depression distinguishing ability indicating that they may be effective biological markers for identifying depression. EEG, fNIRS and machine learning constitute an effective method for classifying depression at the individual level.

## 1. Introduction

Depression is a common mental disorder that seriously affects patients’ social function and daily life. In recent years, enormous pressure has been placed on individuals due to the accelerated pace of life. Under the context of the COVID-19 pandemic, panic and anxiety have increased (Kok et al., 2022). According to the World Health Organization, an estimated 3.8% of the global population has experienced depression, with over 280 million individuals affected by depression (World Health Organization, 2018). Symptoms induced by depression, such as low mood and insomnia, seriously affect the patient’s normal work and life and negatively impact their health. In the worst-case scenario, these symptoms can lead to suicide (Orsolini et al., 2020). Depression is characterized by its persistent existence, high recurrence rate, diverse symptoms, and significant individual differences in treatment efficacy. The detection and diagnosis of depression often present challenges for clinical doctors. So, there is an urgent need to develop objective and accurate diagnostic methods.

Depression diagnosis is usually based on the experience of clinical doctors and the evaluation of depression scales (Tao et al., 2021). However, depression scales have several limitations, such as patients’ denying their symptoms and subjective bias. Objective physiological indicators are beneficial for the diagnosis of depression. Brain functional imaging techniques are now widely used in depression research (Ramasubbu et al., 2016; Herold et al., 2018; Lai, 2019; Kabbara et al., 2022), including functional magnetic resonance imaging (fMRI), functional near-infrared spectroscopy (fNIRS) and electroencephalogram (EEG), etc. Studies have shown that depression may be a mental illness caused by abnormal brain function or structure in the hippocampus (Liu et al., 2013), dorsolateral prefrontal cortex (Grimm et al., 2008), anterior cingulate cortex and posterior cingulate cortex (Yao et al., 2009; Guo et al., 2012). Depression is also related to abnormal brain network topological properties, including global and local connectivity abnormalities. Ye et al. (2015) used resting-state fMRI and the graph theory and found that patients with major depressive disorder (MDD) had higher local efficiency and modularity, compared with the healthy control (HC) group. An EEG study showed that the mild depression group had a larger characteristic path length and lower clustering coefficient than that of the HC group (Li et al., 2020). Brain functional imaging research promotes the understanding of the brain function of depression and lays the foundation for depression diagnosis with neurological indicators.

Among these neuroimaging techniques, fMRI has high spatial resolution, but low time resolution (Arbabshirani et al., 2017). Also, the strong magnetic field environment and strict motion restriction make some subjects feel uncomfortable. EEG has high time resolution, but poor spatial resolution. fNIRS has the same physiological basis as fMRI and can detect neural activity indirectly by measuring hemodynamic signals. fNIRS can provide reasonable time and spatial resolution and has good repeatability and stability (Zhang et al., 2015). The combination of EEG and fNIRS can provide complementary physiological information, and has the characteristics of non-invasiveness (Lacerenza et al., 2020), low cost, easy operation and feasibility for long-term and repeated monitoring (Cai et al., 2020; Zhao et al., 2021). Therefore, this study aims to combine EEG and fNIRS to study the brain function of depression and to establish an automated diagnostic assessment method for depression.

Machine learning (ML) is a research field that enables computers to automatically learn patterns and rules from data (Wu et al., 2021). Combined with neuroimaging data, ML can be used for the diagnosis of patients. Hosseinifard et al. (2013) extracted nonlinear features from four EEG bands and used k-nearest neighbor, linear discriminant analysis, and logistic regression as classifiers to distinguish normal individuals and depression patients, achieving the highest classification accuracy of 83.3%. Another study on depression diagnosis found alpha2 and theta asymmetry had the highest classification accuracy of 88.33% using support vector machine (SVM) (Mahato and Paul, 2020). Although EEG alone has shown considerable diagnostic potential for depression, combining EEG and fNIRS has the potential to further improve diagnostic performance. Al-Shargie et al. (2016) demonstrated that EEG and fNIRS can improve the sensitivity and specificity in detecting psychological stress, achieving an accuracy increase of 3.4% compared to EEG alone and 11% compared to fNIRS alone. Another study extracted different features from EEG, fNIRS, and EEG + fNIRS signals as biomarkers to quantify human mental workload, and fed them to a SVM classifier. The results showed that the hybrid EEG and fNIRS system achieved a significantly higher accuracy (90.9%) compared to either EEG (85.9%) or fNIRS (74.8%) alone (Aghajani et al., 2017). The combined application of EEG and fNIRS in depression diagnosis is still in its early stages, requiring further exploration and validation of the potential biomarkers.

In this study, we simultaneously recorded EEG and fNIRS signals from depression patients and HC subjects during the resting state and analyzed the abnormal brain network characteristics of patients. Studies have found that delta, theta, and alpha bands are closely associated with the pathophysiology and clinical symptoms of depression, making them widely utilized in depression biomarker research (Kang et al., 2020; Ghiasi et al., 2021). Additionally, depression is linked to abnormal brain network topological properties (Ye et al., 2015; Li et al., 2020). Therefore, we extracted brain network properties from these three bands as candidate features. Besides, we extracted asymmetry features of delta, theta and alpha, as well as brain oxygenation entropy, lateralization, and functional connectivity strength as alternative features. By establishing an automated feature selection method and an SVM model, we classified the two groups of subjects. It was expected that the hybrid EEG and fNIRS system would provide better discrimination of depression patients.

## 2. Materials and methods

### 2.1. Participants

A total of 25 patients with major depressive disorder (MDD) and 30 age-, gender-, and education-matched healthy controls were recruited (Table 1). MDD were recruited from the Third People’s Hospital of Foshan, and their clinical severity of depression was evaluated using the 24-item Hamilton Depression Rating Scale (HAMD-24).

**Table 1:** Demographic information for the two groups.

	HC (*n* = 30)	MDD (*n* = 25)	χ^2^/T	*p*
Age	27.43 ± 9.47	28.84 ± 10.70	1.591	0.213
Gender (M/F)	12/18	9/16	0.092	0.761
Education years	13.17 ± 2.65	12.60 ± 2.74	0.033	0.857
HAMD-24	0.40 ± 0.67	19.76 ± 10.37	87.480	<0.001

Data are presented as mean ± standard deviation. A χ^2^ test was used for gender comparison and *T*-tests were used for Age, Education years and HAMD-24 comparison between the two groups.

The inclusion criteria for all participants: age between 16 and 55 years, right-handedness and junior high school education or above. The inclusion criteria for MDD were as follows: (Kok et al., 2022) meeting the diagnostic criteria for depression in the fifth edition of the Diagnostic and Statistical Manual of Mental Disorders (DSM-V) based on clinical psychiatric interviews (World Health Organization, 2018), being diagnosed as MDD by two attending psychiatrists, and (Orsolini et al., 2020) without psychiatric medication treatment in the past 2 weeks. The exclusion criteria for MDD: (Kok et al., 2022) the presence of neurological or severe physical disease (World Health Organization, 2018), a history of drug abuse or dependence; (Orsolini et al., 2020) the presence of other psychiatric disorders, such as bipolar disorder, obsessive–compulsive disorder, schizophrenia, or personality disorders. For HC, exclusion criteria include a personal or family history of psychiatric disorders.

All participants were informed of the experimental procedures and provided written informed consent. This study was approved by the Ethics Committee of the Third People’s Hospital of Foshan and Foshan University, and all procedures performed in this study were in accordance with the committee’s ethical guidelines.

### 2.2. Experimental design and data acquisition

We adopted a resting-state paradigm. During the experiment, participants were seated comfortably in a quiet room and instructed to relax with their eyes closed but remain awake. EEG and fNIRS signals were recorded simultaneously for 6 min. All data collection was conducted between 10 a.m. and 4 p.m., with room temperature maintained at 23 ± 2°C. Regarding the data acquisition time, we completed all data collection between July 2022 and March 2023. No dropouts occurred during the entire data collection process.

**Figure 1:**
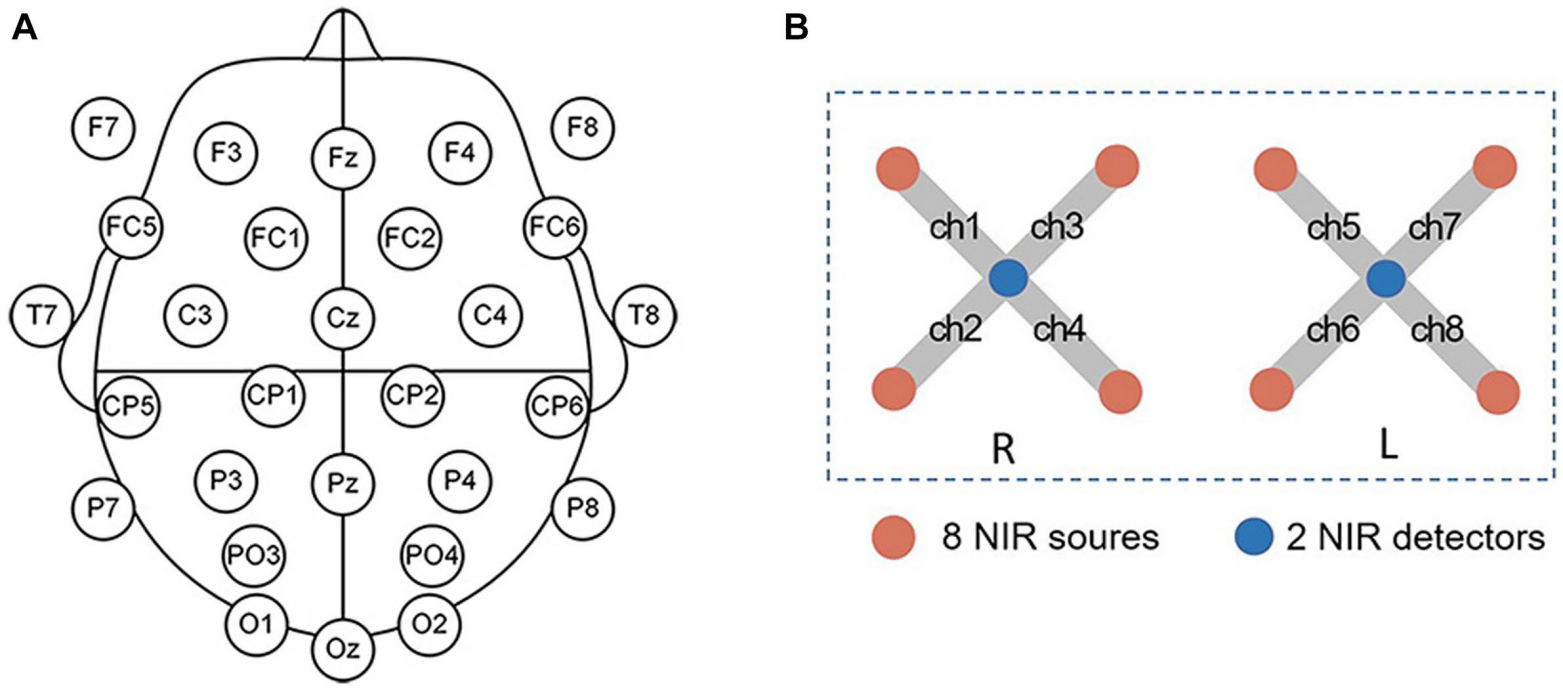
**(A)** The EEG electrode positions. **(B)** fNIRS channel positions. L is for the left hemisphere, and R is for the right hemisphere.

A wireless EEG acquisition system with 32 channels (NeuSen W, Neuracle, Changzhou, China) was used for the EEG recordings, with REF as the reference electrode and GND as the ground electrode. The EEG data from 28 electrodes were recorded according to the standard 10–20 system (Fz, F3, F4, F7, F8, FC1, FC2, FC5, FC6, Cz, C3, C4, T7, T8, CP1, CP2, CP5, CP6, Pz, P3, P4, P7, P8, PO3, PO4, Oz, O1, O2, Figure 1). The EEG sampling rate was 500 Hz and the impedance for all electrodes was kept below 30 kΩ during the experiment.

The fNIRS signals were recorded by using a device from Artinis, OctaMon. The device comprised two detectors and eight emitters (light sources) forming eight fNIRS channels (Figure 1). OctaMon emits near-infrared light at 760 and 850 nm, and can measure concentration changes of oxygenated and deoxygenated hemoglobin (Δ[HbO2] and Δ[Hb]) based on the modified Lambert–Beer’s law. The source-detector distance was set as 35 mm and the differential path length factor was set as 6. During the experiment, the fNIRS probe was aligned with the midline and placed on the participant’s forehead, with the probe secured in place using a bandage to prevent movement and minimize any external light interference. The fNIRS signals were acquired at a sampling rate of 10 Hz.

### 2.3. Data preprocessing

In this study, signal processing and analysis were performed using EEGLAB and MATLAB. For EEG data preprocessing, EEG signals from T7 and T8 were first removed due to the influence of muscle activity. Then, a 50 Hz notch filter was used to eliminate the powerline interference followed by a bandpass filter of 0.5–30 Hz. After the initial 10-s EEG data were discarded, the filtered EEG data were then re-referenced to the average reference and then segmented into 40-s epochs. Epochs with absolute values exceeding ± 100 μV were excluded automatically. After visual inspection of all EEG data, the first four 40-s epochs (26*20000*4) were artifact-free and were used to extract the delta (0.5–4 Hz), theta (4–8 Hz), and alpha (8–13 Hz) bands for subsequent analyses.

For fNIRS data preprocessing, a second-order bandpass Butterworth filter (0.01–0.1 Hz) was first applied to eliminate low-frequency noise and baseline drift (Sasai et al., 2011). The fNIRS signals may appear to drift over time. A third-order polynomial fit was used to remove drift, followed by wavelet denoising (Bornhövd et al., 2002). Compared with Δ[Hb], Δ[HbO2] has a higher signal-to-noise ratio (Strangman et al., 2002), therefore only HbO2 data were analyzed in this study. For each participant, after the initial 30-s data were discarded, 3-min stable Δ[HbO2] signals were selected for subsequent analysis (Pang et al., 2022).

### 2.4. EEG network analysis

To analyze the abnormal brain network characteristics of patients, the phase locking value (PLV) (Lachaux et al., 1999) was used to calculate the functional connectivity between all electrode pairs of the EEG time series. The range of PLV is between 0 (no synchronization) and 1 (complete synchronization). The instantaneous phase (*Ψ*) of each signal was obtained using the Hilbert transform, and the PLV between two signals *x* and *y* was defined by equation (1):


(1) $$PLV=|\frac{1}{N}\overset{n}{\underset{t=1}{\sum }}e^{i\left(\Psi_{x}\left(t\right)-\Psi_{y}\left(t\right)\right)}|$$

Where *Ψ_x_* and *Ψ_y_* and are the instantaneous phases for signals *x* and *y* at time *t*, and *N* represents the window size used to calculate the PLV.

PLV was first applied to each 40-s epoch which is a stable period to calculate the brain’s resting state functional connectivity (Kabbara et al., 2017), resulting in four 26 × 26 adjacency matrices for each subject. Then, these four 26 × 26 PLV matrices were averaged to obtain the final PLV matrix to calculate the network properties.

The graph theory was used to analyze the small-world properties. The PLV matrix was first converted to the corresponding binary network through sparseness with different thresholds. The choice of the threshold is critical for constructing the network. Low threshold values lead to dense connections while high threshold values lead to sparse networks (Achard et al., 2006). In this study, we used a proportional threshold to remove weak connections from the PLV matrix. The PLV matrix was sorted from largest to smallest, and connections larger than the threshold were set to 1, while the rest were set to 0.1 means that an edge was present between two channels, otherwise, no edge was present. If a threshold value was set as 0.3, the first 70% of PLV matrix values are designated as 1, while the remaining values are designated as 0. We chose a threshold range of 0.2–0.5, with a stepsize of 0.01, to ensure the comparison of network properties between the two groups under the same connection. After the sparse binary network was constructed, six brain network properties (clustering coefficient: C, characteristic path length: L, global efficiency: GE, local efficiency: LE, transitivity: T, and modularity: M) were calculated using the Brain Connectivity Toolbox (BCT) (Rubinov and Sporns, 2010).

### 2.5. Feature extraction and machine learning

#### 2.5.1. AUCs of EEG network properties

To avoid the influence of threshold on EEG network property analyses, we used the area under the curve (AUC) (Zhang et al., 2015) of six brain network properties at the threshold range of 0.2–0.5 as alternative features. AUC is the area between the curve of topological properties and the *X*-axis by the numerical integration method, and it can provide a summary scalar for the corresponding network property (Shi et al., 2021). AUC can avoid the influence of a single threshold and research has demonstrated its sensitivity in detecting topological changes associated with brain disorders (Zhang et al., 2015; Han et al., 2020). Thus, in this study, we computed the AUCs in the delta, theta, and alpha bands as candidate features.

#### 2.5.2. EEG inter-hemispheric asymmetry

For EEG signals at each band, we used the relative power difference between each electrode and its symmetrical electrode to obtain the inter-hemispheric asymmetry as a candidate feature. Specifically, we used the Welch’s Hanning method to compute the power spectrum for the EEG signal at each electrode. In the Welch method, the time series were divided into 8.192-s segments (50% overlap), and the modified periodograms of all segments were averaged to obtain the power spectral density S for each frequency band. Equation (2) was used to calculate the relative power of the target frequency band, where *f_1_* and *f_2_* represent the lowest and highest frequencies in the band, respectively. For example, *f_1_* was 0.5 Hz and *f_2_* was 4 Hz for delta.


(2) $$R_{ch1}=\frac{\sum_{f_{1}}^{f_{2}}S_{ch1}}{\sum_{0.5Hz}^{30}S_{ch1}}\;L_{ch2}=\frac{\sum_{f_{1}}^{f_{2}}S_{ch2}}{\sum_{0.5Hz}^{30}S_{ch2}}$$

(3) $$Asymm\left(ch1,ch2\right)=\frac{R_{ch1}-L_{ch2}}{R_{ch1}+L_{ch2}}$$

Equation (3) provides an example to compute the asymmetric score of ch1 and ch2, where *R_ch1_* represents the relative power of the EEG signal at a specific right electrode, while *L_ch2_* represents the relative power of the EEG signal at its corresponding symmetrical left electrode. Therefore, the electrode pairs included: F4-F3, F8-F7, FC2-FC1, FC6-FC5, C4-C3, CP2-CP1, CP6-CP5, P4-P3, P8-P7, PO4-PO3, and O2-O1, totaling 11 electrode pairs.

#### 2.5.3. HbO2 sample entropy

In this study, we used the Sample Entropy (SampEn) (Richman and Moorman, 2000) to calculate the complexity of the HbO2 signal as candidate features. Specifically, for an N-point time series signal, its SampEn is calculated using the following equations:


(4) $$SampEn\left(m,r,N\right)=-\ln\left[\frac{U^{m+1}\left(r\right)}{U^{m}\left(r\right)}\right]$$

(5) $$U^{m}\left(r\right)={\left[N-m\tau \right]}^{-1}\overset{N-m\tau }{\underset{i=1}{\sum }}C_{i}^{m}\left(r\right)$$

(6) $$C_{i}^{m}\left(r\right)=\frac{B_{i}}{N-\left(m+1\right)\tau }$$

where *m* represents the embedding dimension, *r* is the tolerance factor for the difference between two subsequences, and *τ* represents the time delay. *B_i_* = number of *j* where *d |x_i_, x_j_|* ≤ *r, x_i_ =* (*x_i_, x_i + τ_,…x_i + (m-1)τ_*), *x_j_ =* (*x_j_, x_j + τ_,…x_j + (m-1)τ_*)*, i* ≤ *j* ≤ *N-mτ, j ≠ i.* In this study, we set *τ* = 2, *m* = 2 and *r* = 0.2*SD (Richman and Moorman, 2000).

#### 2.5.4. HbO2 functional connectivity

In this study, we divided the eight fNIRS channels into four regions (roi1: ch1 and ch3, roi2: ch2 and ch4, roi3: ch5 and ch7, roi4: ch6 and ch8). The HbO2 at each region was calculated as the average of the two channels within that region. We then calculated the Pearson correlation coefficients between the HbO2 time series from two regions and performed Fisher-Z transformation to improve the normality (Rogers et al., 2007). The transformed Z values were defined as the functional connectivity strength between regions and used as candidate features.

#### 2.5.5. HbO2 laterality index

As referenced from previous studies (Wang et al., 2023), we also computed the laterality index (LI) of HbO2 as an alternative feature using Equation (7). Here, ΔoxyR and ΔoxyL represent HbO2 concentration changes in the right (four right channels were averaged) and left (four left channels were averaged) forehead, respectively. The range of LI is between −1 and 1, where a positive LI indicates greater activity in the right forehead compared to the left forehead, and a negative LI indicates greater activity in the left forehead compared to the right forehead.


(7) $$LI=\frac{\sum \left[\left(\Delta \mathrm{oxyR}-\min\left(\Delta \mathrm{oxyR}\right)\right)-\left(\Delta \mathrm{oxyL}-\min\left(\Delta \mathrm{oxyL}\right)\right)\right]}{\sum \left[\left(\Delta \mathrm{oxyR}-\min\left(\Delta \mathrm{oxyR}\right)\right)+\left(\Delta \mathrm{oxyL}-\min\left(\Delta \mathrm{oxyL}\right)\right)\right]}$$

#### 2.5.6. Feature selection and SVM model

After extracting the above candidate features, we obtained a 55 × 67 two-dimensional feature matrix. Before classification, we normalized these features within the range of 0 and 1 using the following equation (8):


(8) $$X^{\prime }=\frac{X_{i}-X_{\min}}{X_{\max}-X_{\min}}$$

where *X*’ is the normalized feature value, *X* is the original feature value, *X_max_* and *X_max_* are the maximum and minimum values of the feature *X*, respectively.

Among these alternative features, there were many redundant features. Feature selection can reduce the number of input variables for the classifier, which can effectively improve the model recognition (Mumtaz et al., 2018). The least absolute shrinkage and selection operator (LASSO) regression (Tibshirani, 1997) was used to perform feature selection on the input feature matrix. LASSO penalizes the coefficients of regression variables so that some feature variable parameters shrink to 0. The LASSO method uses L1 norm regularization to select variables, retaining only the features with non-zero coefficients after the shrinkage process. This feature selection not only retained useful feature information as much as possible but also reduced the complexity of the model. Suppose 𝐗∈R^𝑁×𝑚^ consists of 𝑁 samples containing 𝑚 features, 𝐲∈R^𝑁^ is the response vector, and 𝜷∈R^𝑚^ is the vector of regression parameters. LASSO is mathematically represented as follows (Liu et al., 2013):


(9) $$\beta^{*}=argmin_{\beta }{\left||y-X\beta |\right|}_{2}^{2}+\lambda {\left|\left|\beta \right|\right|}_{1}$$

where 𝜷* is the optimal solution of the problem, ||﹒||_1,_ ||﹒||_2_ represent the L1 norm and L2 norm respectively, *λ* ≥ 0 is the penalty parameter. The L1 regularization imposes a penalty on the regression coefficients 𝜷, leading to automatic shrinkage of smaller absolute values of 𝜷 components toward 0. Therefore, a sparse optimal solution can be obtained, and as the penalty parameter increases, the solution becomes sparser. In the feature selection process, we employed a 10-fold cross-validation strategy to identify the best performing feature subset within the training set, which served as the input variables for the SVM model.

SVM was used to construct the classification model (Cortes and Vapnik, 1995). SVM has unique advantages in solving small sample, nonlinear, and high-dimensional feature recognition problems and has largely overcome problems such as the “curse of dimensionality” and overfitting (Erfani et al., 2016). SVM constructs a hyperplane that separates the target class from the other classes by minimizing the error and maximizing the margin, which is the distance between the hyperplane and the closest sample points from each class (Cortes and Vapnik, 1995). Based on the optimal feature set selected by the LASSO regression, we established an SVM model and optimized the kernel function parameters (C = 1, kernel = ‘linear’). We used the Leave-One-Out Cross-Validation (LOOCV) (Cawley and Talbot, 2004) to evaluate the performance of the model, where one participant was used as the test sample while the remaining participants served as the training samples. We performed backward feature elimination during the LOOCV process and identified the subset with the highest recognition accuracy as the final classification feature. Meanwhile, Accuracy, Precision, and Recall were used to evaluate the generalization performance of our model.

**Figure 2:**
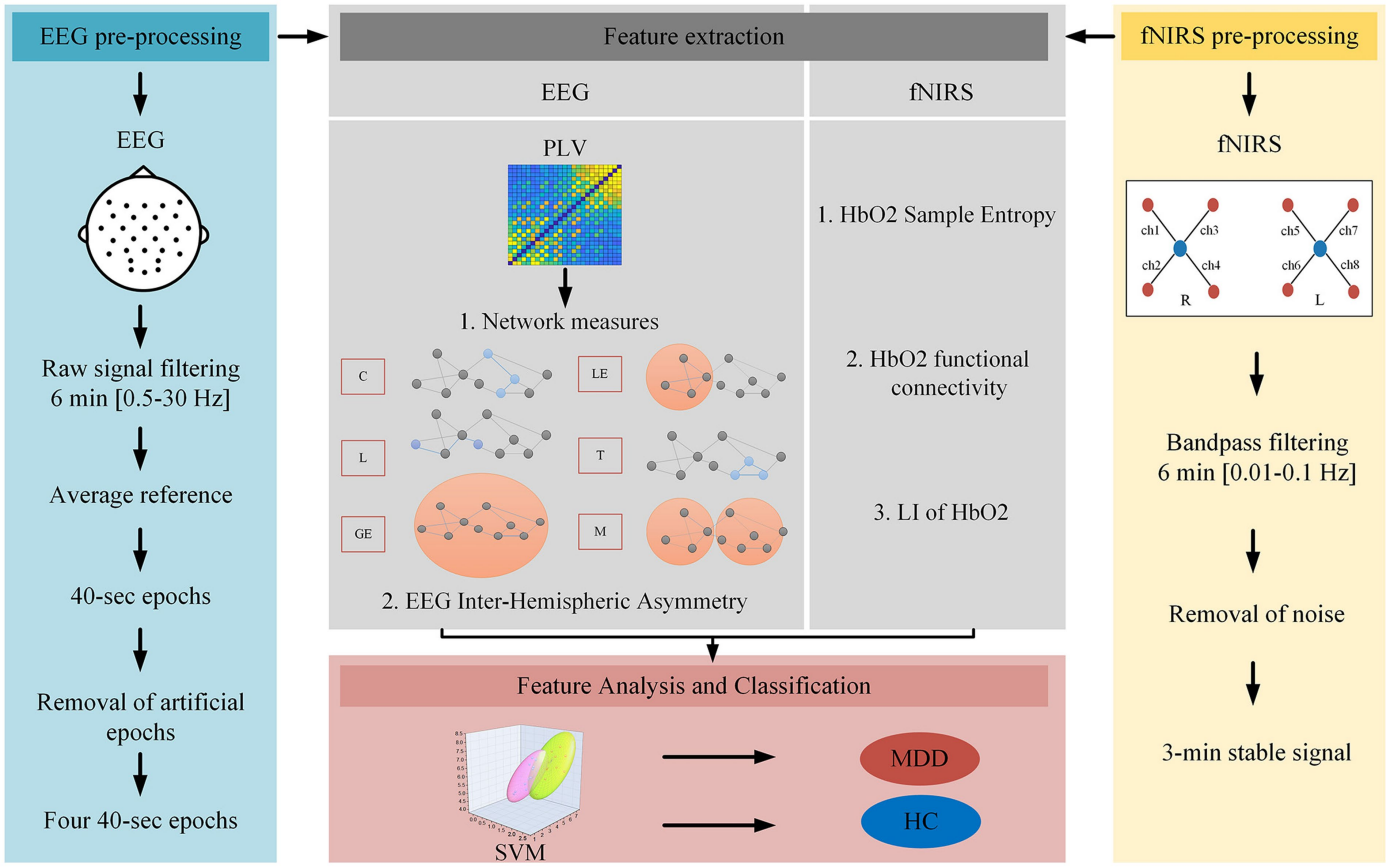
EEG and NIRS data processing and analyses flowchart.

Figure 2 shows the flow chart for processing and analyzing EEG and NIRS data in this study.

### 2.6. Statistical analyses

In our study, Shapiro–Wilk tests were used to determine whether the data were normally distributed. We employed the independent samples *t*-test or the Mann–Whitney *U* test to compare the measurement data between the two groups, and all *t*-tests were corrected by FDR (False Discovery Rate). The effect size was calculated using the GPower software, and the level of statistical significance was set at 0.05. Python was used for automatic feature selection and classification modeling.

## 3. Results

### 3.1. EEG network results

**Figure 3:**
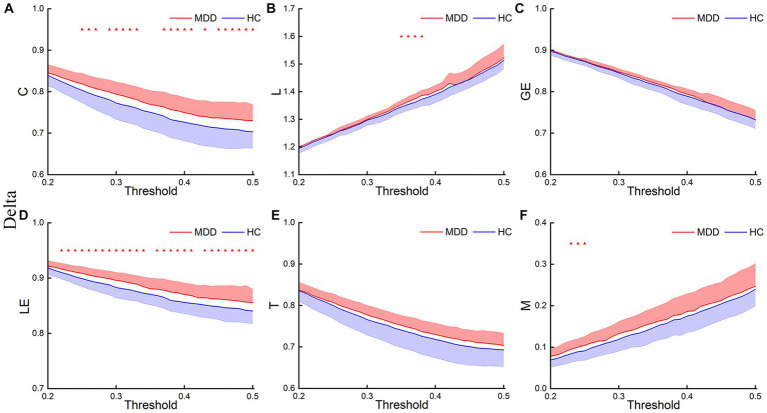
Delta network topological properties. The mean C **(A)**, L **(B)**, LE **(C)**, GE **(D)**, T **(E)**, and M **(F)** for the two groups under each threshold. The shaded part indicates the standard error (SEM) (*p* < 0.05, FDR correction).

**Figure 4:**
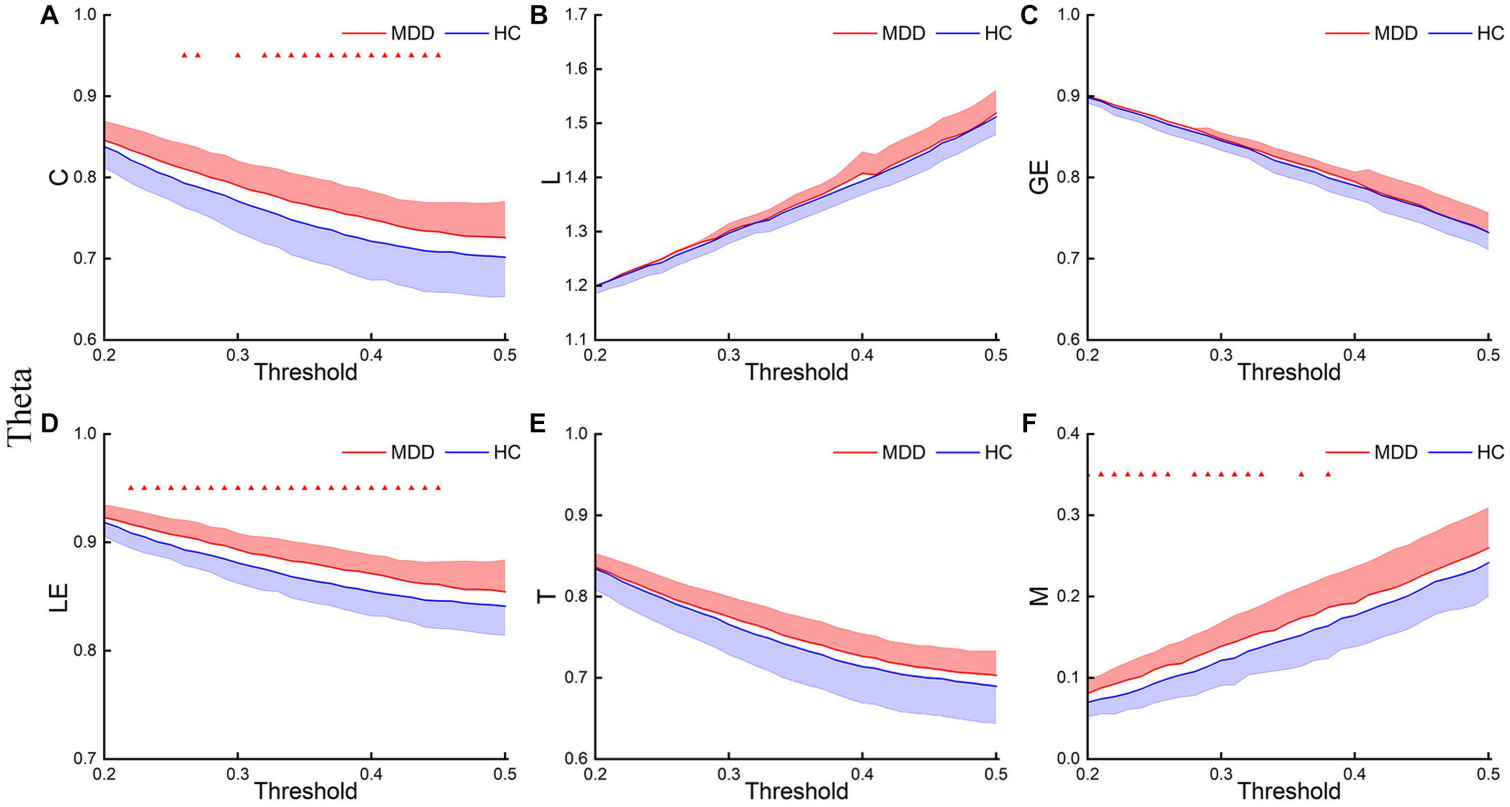
Theta network topological properties. The mean C **(A)**, L **(B)**, LE **(C)**, GE **(D)**, T **(E)**, and M **(F)** for the two groups under each threshold. The shaded part indicates the SEM (*p* < 0.05, FDR correction).

**Figure 5:**
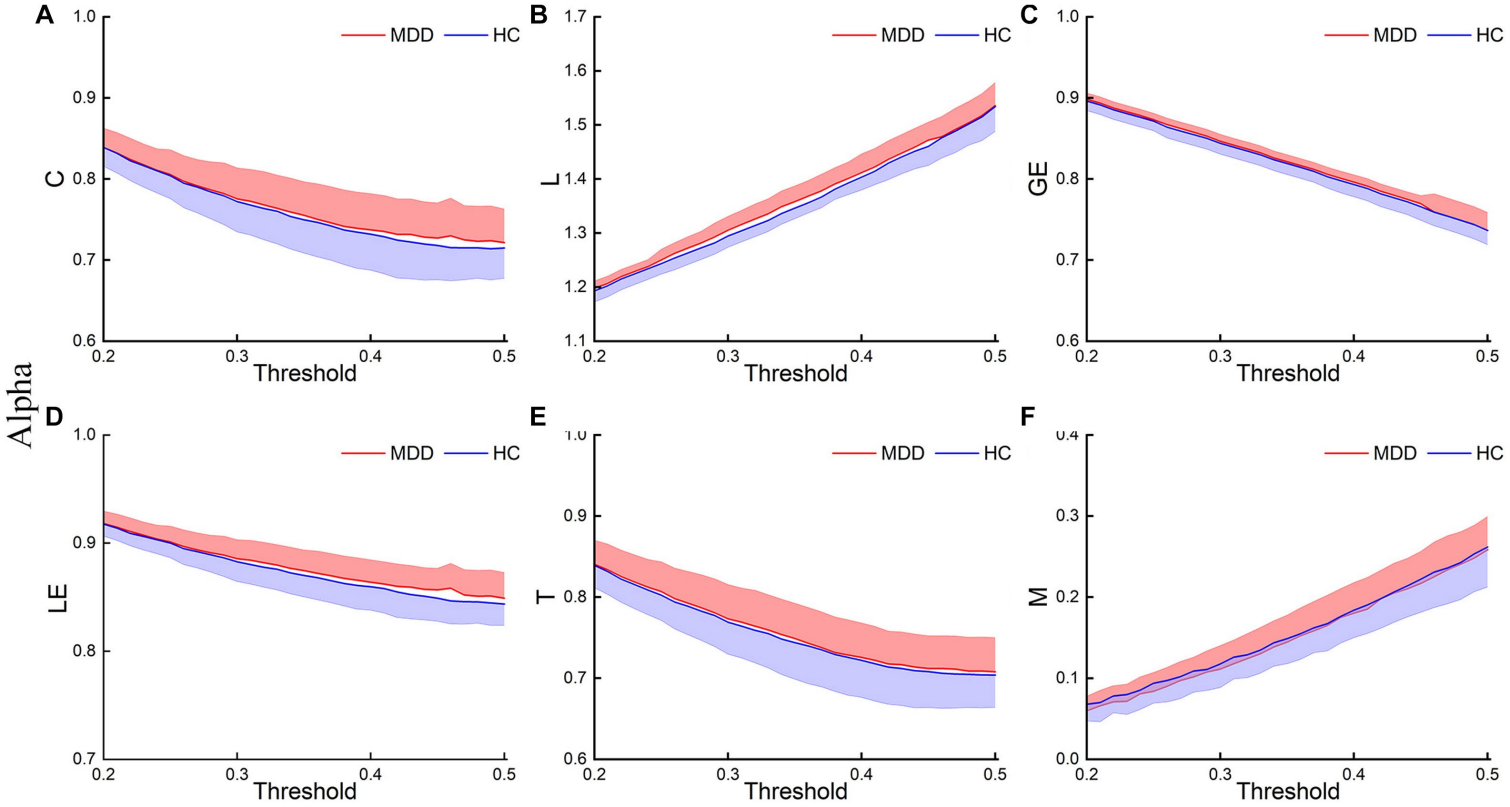
Alpha network topological properties. The mean C **(A)**, L **(B)**, LE **(C)**, GE **(D)**, T **(E)**, and M **(F)** for the two groups under each threshold. The shaded part indicates the SEM (*p* < 0.05, FDR correction).

Figures 3–5 show the network topological property trend along the threshold in different EEG bands for the two groups of subjects. Along with the threshold increase, some connections got lost, leading to a decrease in C. This loss of connections also resulted in connections between node pairs passing through more nodes, leading to an increase in L. The global and local efficiency as well as transitivity also decreased with increasing thresholds, while modularity increased with increasing thresholds.

This study first analyzed the abnormal brain network characteristics of patients. *T*-test results showed that, in the delta and theta bands, C and LE of MDD increased compared to the HC, and L and M of MDD also increased at certain thresholds (Figures 3, 4). There was almost no significant difference in GE and T between the two groups. And there was no significant difference in brain network properties between the two groups in the alpha band.

**Figure 6:**
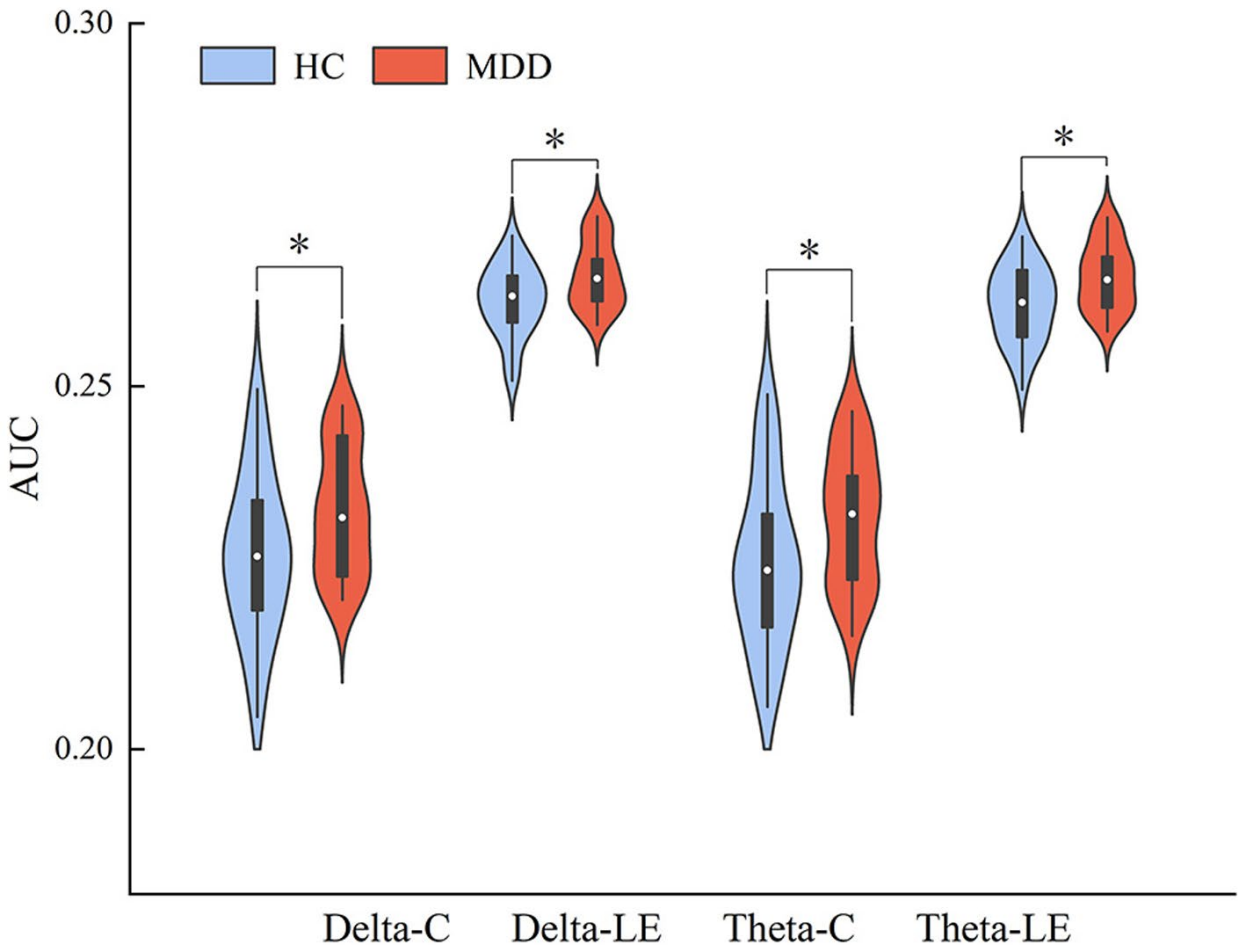
AUC indicators that are significantly different between the two groups, * indicates *p* < 0.05, ** indicates *p* < 0.01 (FDR correction).

We also compared the AUCs of the six network properties between the two groups. The *T*-test results showed significant differences in the AUCs of C and LE in the delta and theta bands between the two groups (Figure 6). The results were as follows: delta-C (*p* = 0.032, Cohen’s d = 0.61) and delta-LE (*p* = 0.015, Cohen’s *d* = 0.76); theta-C (*p* = 0.032, Cohen’s *d* = 0.60) and theta-LE (*p* = 0.014, Cohen’s *d* = 0.79). The *p*-values were corrected using FDR correction.

### 3.2. Classification results

**Figure 7:**
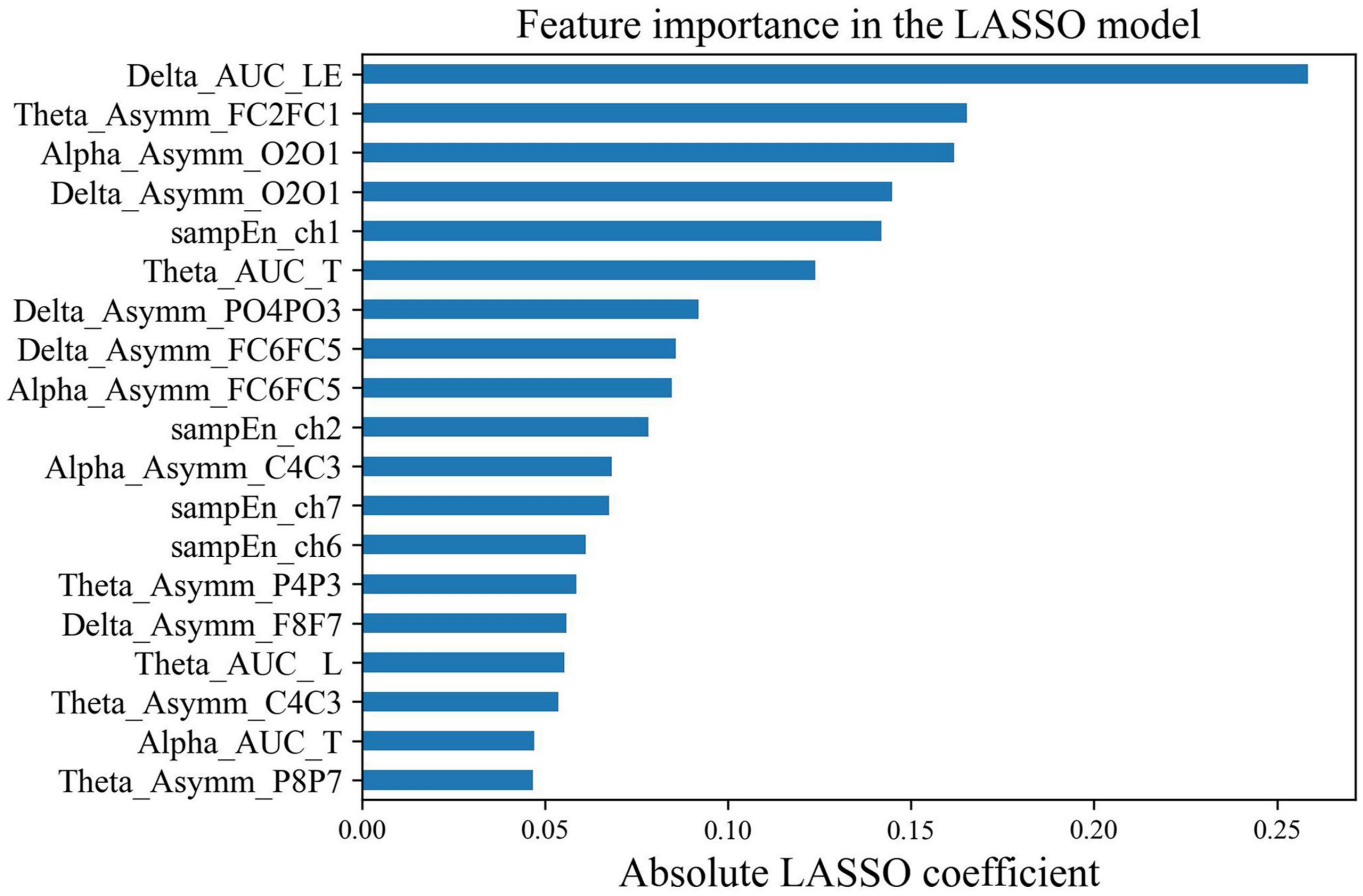
Feature importance in the (EEG and fNIRS) LASSO Model.

After LASSO feature selection, we selected the optimal feature subset, that is, 19 EEG and fNIRS features shown in Figure 7. In order to obtain reliable biomarkers for depression, we also performed t-tests to analyze the differences in the top six features after LASSO between the two groups. The results showed significant differences between the two groups in local efficiency in the delta band (*p* = 0.015, Cohen’s *d* = 0.76), asymmetry of FC1-FC2 in the theta band (*p* = 0.003, Cohen’s *d* = 0.84), and HbO2 sample entropy at ch1 (*p* = 0.016, Cohen’s *d* = 0.82) (Table 2).

**Table 2:** *T*-test results for the top six features between the two groups.

No	Features	*T*-value	*P*-value	Cohen’s d
1	Delta_AUC_LE	−2.787	0.015	0.76
2	Theta_Asymm_FC2FC1	−3.162	0.003	0.84
3	Alpha_Asymm_O2O1	1.063	0.293	0.28
4	Delta_Asymm_O2O1	0.710	0.367	0.24
5	SampEn_ch1	−2.488	0.016	0.67
6	Theta_AUC_T	−1.102	0.275	0.30

Negative *T*-values indicate higher values for the patient group.

**Figure 8:**
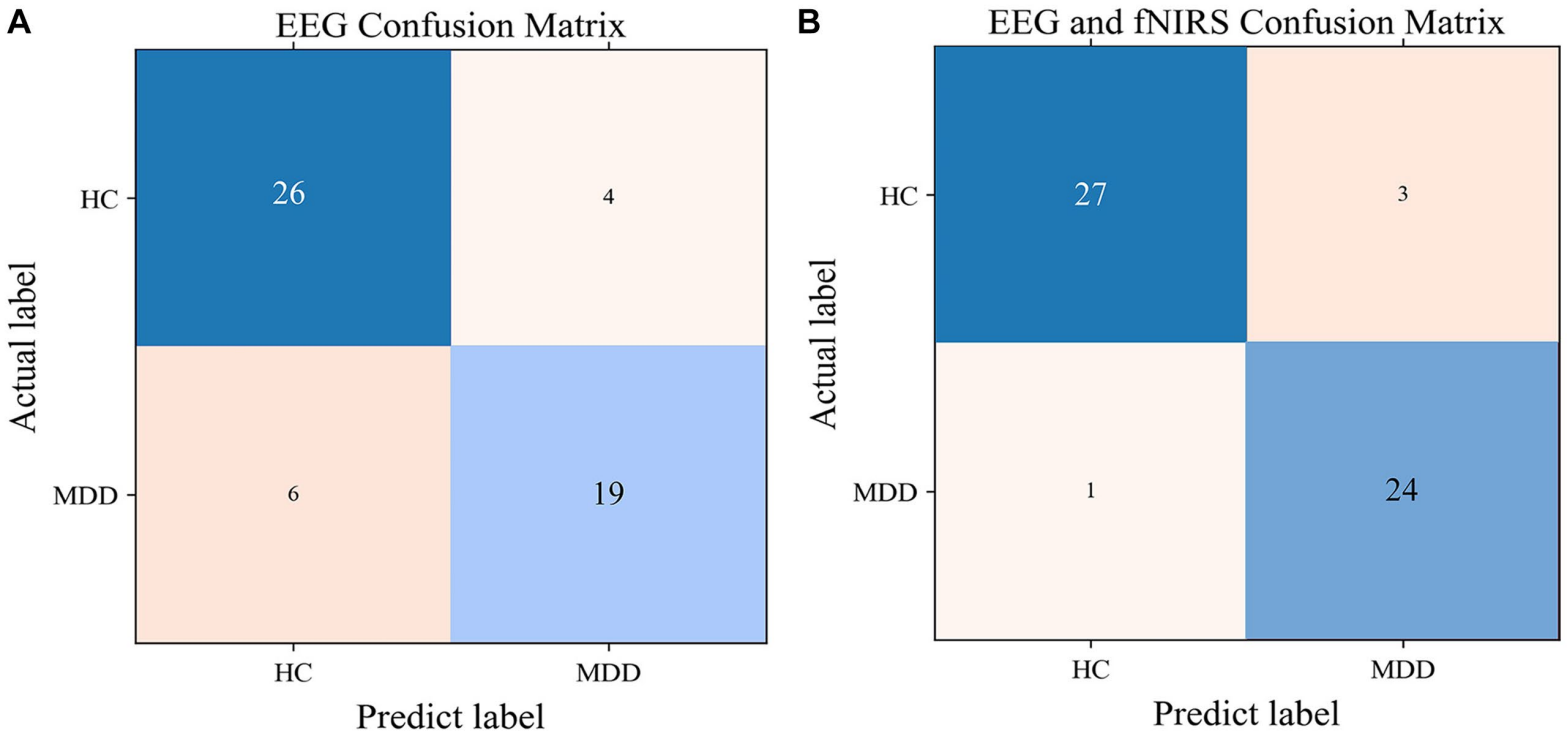
The confusion matrix for the SVM model with EEG features **(A)** and with EEG and fNIRS features **(B)**.

**Figure 9:**
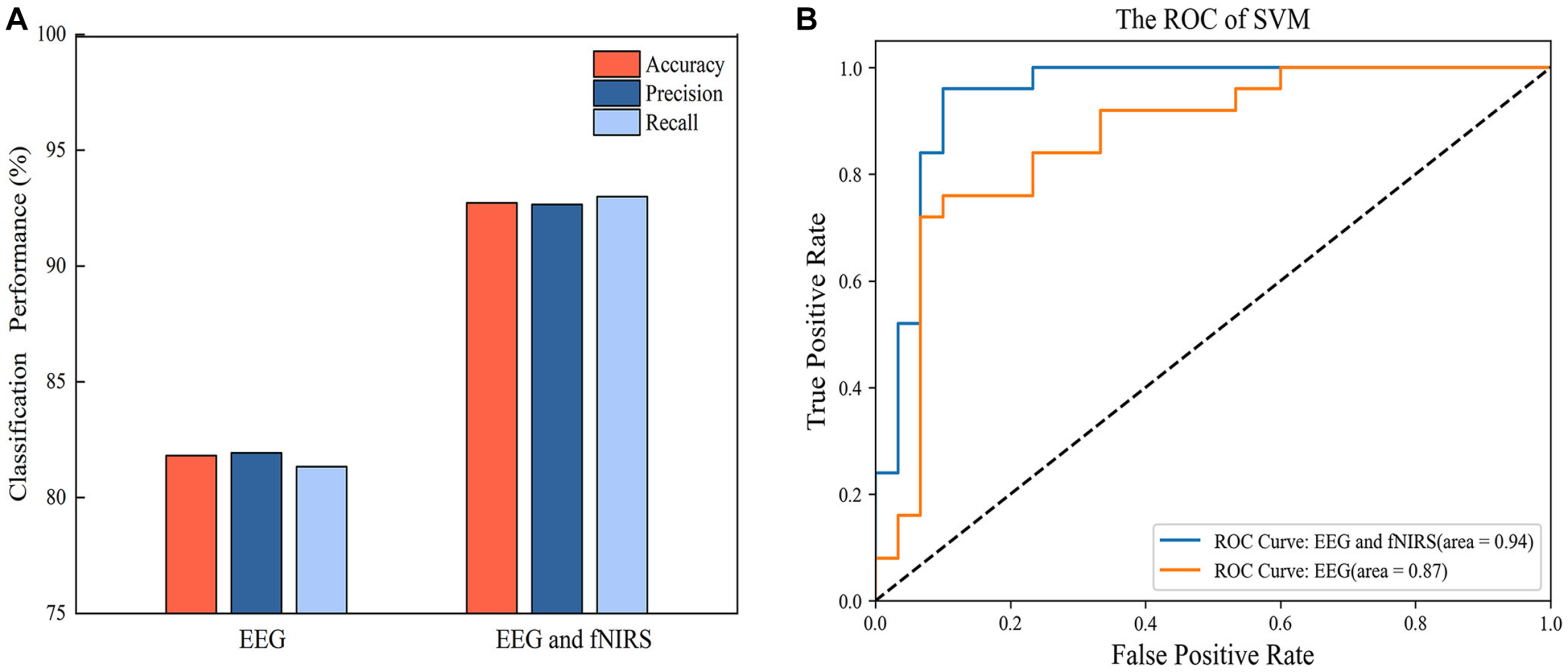
**(A)** The classification performance of SVM with EEG features and hybrid EEG and fNIRS features. **(B)** The ROC curve of the SVM models.

SVM models were built with single EEG features and with hybrid EEG and fNIRS features, separately. The confusion matrixes for the two SVM models are shown in Figure 8, and the classification Accuracy, Precision, and Recall for the two SVM models are shown in Figure 9A. Using EEG features, the SVM classification accuracy was 81.8%, with the precision of 81.9% and the recall of 81.3%. But when mixed in fNIRS features, the calculated average accuracy was 0.927 with a standard deviation of 0.036, precision and recall of 92.7 and 93.0%, respectively. The receiver operator characteristic (ROC) curves of the SVM models are presented in Figure 9B, with AUC = 0.87 for EEG features alone, and AUC = 0.94 for hybrid EEG and fNIRS features.

## 4. Discussion

This study examined the resting-state functional network properties of MDD patients and attempted to establish an automatic identification method for MDD using EEG and fNIRS features. The results showed that MDD had higher clustering coefficients and local efficiency in the delta and theta bands. Through automated feature selection, we constructed an SVM model with both EEG and fNIRS features that achieved high classification rates of 92.7% for the two groups of subjects.

Our results revealed that MDD exhibited higher LE, C, and M in the delta and theta bands. The increased clustering coefficient of patients’ brain networks suggests an increase in the local short connections, indicating high local clustering phenomena. Furthermore, patients’ local efficiency was significantly higher than that of the HC group, which may represent a decrease in information processing efficiency between distant brain regions (Rocca et al., 2016). These results indicate that some local regions in the brain network of MDD have abnormal neural connections, leading to a tendency for the brain network to become more tightly and locally connected and separate globally in certain bands. Our findings are consistent with those of an fMRI study (Ye et al., 2015) but differ from those of Li et al. (2020) and Leistedt et al. (2009). Leistedt et al. found that the path length in the theta and delta frequency bands was significantly reduced in acute depressive patients. This difference may be due to different analysis methods and patients’ clinical manifestations.

The feature importance ranking showed that the local efficiency in the delta band was the most distinguishing factor in identifying depression, followed by the FC1-FC2 hemisphere asymmetry in the theta band, the O2-O1 asymmetry in the alpha band, the O2-O1 asymmetry in the delta band, and the HbO2 SampEn of the right forehead in sequence. Among them, there were significant differences between the two groups in the local efficiency of the delta band (*p* = 0.015, Cohen’s *d* = 0.76), the FC1-FC2 hemisphere asymmetry in the theta band (*p* = 0.003, Cohen’s *d* = 0.84), and the HbO2 SampEn at ch1 (*p* = 0.016, Cohen’s *d* = 0.67). Currently, many studies have shown that the frontal asymmetry in the alpha and theta bands can serve as a biomarker for evaluating depressive disorders (Hinrikus et al., 2009; Duan et al., 2020; Mahato and Paul, 2020; Kang et al., 2021). Our research confirmed that the frontal asymmetry in theta band of depressed patients was greater than that of healthy individuals. Under the joint verification of machine learning classification and statistical analyses, this feature can be used to distinguish depressed patients from healthy individuals. Furthermore, the forehead HbO2 SampEn was higher in depressed patients than that in healthy subjects, indicating that patients’ neural activity in this region is more complex during the rest state (Perpetuini et al., 2020). The features selected by our machine learning model and the significant group differences in statistical analyses are not entirely consistent. Low *p*-values do not necessarily indicate good single-subject-level prediction performance, further emphasizing the need for AI involvement in precision medicine (Bonkhoff and Grefkes, 2022).

In this study, we used EEG with another complementary neuroimaging technique, fNIRS. The incorporation of fNIRS features improved the recognition accuracy of the SVM model by 10.9%, demonstrated the advantages of the joint EEG and fNIRS signals in identifying depressive disorders. EEG and fNIRS measure the electrophysiological and hemodynamic signals of brain activity, respectively, and their combination can provide richer information and enhance detection accuracy. This has also been confirmed in other studies. For example, Li et al. achieved a higher classification accuracy (91.02%) by integrating EEG and fNIRS features in a left and right hand movement task compared to using EEG alone (85.64%) (Li et al., 2017). Al-Shargie et al. demonstrated that the fusion of EEG and fNIRS features could enhance the sensitivity and specificity in assessing mental stress. The accuracy of the fusion method improved by 3.4% compared to EEG alone and by 11% compared to fNIRS alone (Al-Shargie et al., 2016). We developed an SVM model that incorporated mixed EEG and fNIRS features, achieving a classification accuracy of 92.7%. The notable performance of our model might be attributed to two key factors. Firstly, we employed a non-linear phase analysis method (PLV) to construct brain networks, enabling a better capture of the complex temporal dynamics of EEG signals (Lachaux et al., 1999). Secondly, the combination of LASSO and SVM for feature selection effectively addressed the challenges of small sample size and high-dimensional data.

The hybrid EEG and fNIRS is a safe and well-tolerated method of data acquisition for subjects with low environmental requirements. Throughout the entire experiment, no subjects dropped out. This makes the combination method great potential for clinical application or routine screening of depression. Moreover, the development of wireless and wearable EEG and fNIRS devices has made our approach more portable and cost-effective (Buckova et al., 2020; Jeong et al., 2022; Yu et al., 2022), which facilitates screening in high-risk populations at schools or communities.

This study also has some limitations that need to be addressed. Firstly, the sample size was small. However, the effect size demonstrated that the differences in network properties and features between the two groups were reliable. In future research, we will recruit more patients to further validate our method, taking into consideration various factors such as age, gender, and severity level. Secondly, we employed a simple feature-level fusion of EEG and fNIRS features. However, this fusion strategy may overlook the complex relationship between EEG and fNIRS. In the future, we will explore high-level fusion strategies, such as deep learning-based methods or convolutional neural networks, to better leverage the potential information of EEG and fNIRS.

## 5. Conclusion

This study found that patients with depression have higher clustering coefficients and local efficiency in the delta and theta bands, indicating brain network separation and local clustering characteristics. Our study also suggests that the brain network local efficiency in the delta band, hemispheric asymmetry in the theta band and brain oxygen sample entropy features may serve as biological markers for identifying depression. By optimizing hybrid EEG and fNIRS features, we built a machine learning-based individual-level depression diagnosis model, achieving an accuracy of 92.7%. The advantages of hybrid EEG and fNIRS allow our depression recognition model great potential for use.

## Data availability statement

The datasets generated for this study are available on request to the corresponding author.

## Ethics statement

The studies involving humans were approved by the Ethics Committee of the Third People’s Hospital of Foshan and Foshan University. The studies were conducted in accordance with the local legislation and institutional requirements. The participants provided their written informed consent to participate in this study. Written informed consent was obtained from the individual(s) for the publication of any potentially identifiable images or data included in this article.

## Author contributions

LY, GX, JS, and JL: conceptualization. LY, GX, KW, and GS: methodology. YZ: software. JL: validation. LY, ZL, and CZ: formal analysis. GS and HJ: investigation. XL: resources. LY, GX, ZL, XL, BL, HJ, and WL: data curation. LY: writing—original draft. JS and CZ: writing—review and editing. GS: visualization. WL and ZH: supervision. JS: funding acquisition. All authors have read and agreed to the published version of the manuscript.

## Funding

This research was funded by the National Natural Science Foundation of China (32000980 and 82171533), the Guangdong Basic and Applied Basic Research Foundation (2022A1515140142, 2019A1515110427, and 2020B1515120014), the Science and Technology Program of Guangzhou (202103000032), the Key Laboratory Program of Guangdong Higher Education Institutes (2020KSYS001), the project of Foshan Science and Technology Bureau (2220001004473), and the project of Foshan Health Bureau (20230200).

## Conflict of interest

The authors declare that the research was conducted in the absence of any commercial or financial relationships that could be construed as a potential conflict of interest.

## Publisher’s note

All claims expressed in this article are solely those of the authors and do not necessarily represent those of their affiliated organizations, or those of the publisher, the editors and the reviewers. Any product that may be evaluated in this article, or claim that may be made by its manufacturer, is not guaranteed or endorsed by the publisher.
