# Supplementary material for: Automatic depression diagnosis through hybrid EEG and near-infrared spectroscopy features using support vector machine
Source: Front Neurosci. 2023 Aug 24;17:1205931. doi: 10.3389/fnins.2023.1205931 (PMC10483285; doi:10.3389/fnins.2023.1205931)
Supplement: Supplementary file 1 [file Data_Sheet_1.docx]

Supplementary Material

Article Title

# Classification Performance of fNIRS Features

The classification performance obtained from the model with only fNIRS features was lower (accuracy: 69%, precision: 0.68, recall: 0.68, F1 score: 0.68) compared to models with EEG features or with hybrid EEG and fNIRS features. We acknowledge that there is still room for improvement before practical application. As a result, we have not included these classification results in the main text. We added the classification performance with only fNIRS features in the supplementary material.


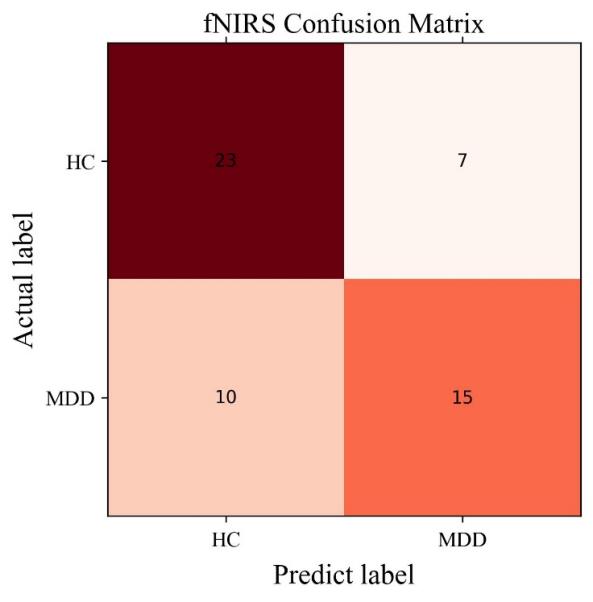


**Figure 1.** The confusion matrix for the SVM model with only fNIRS features.


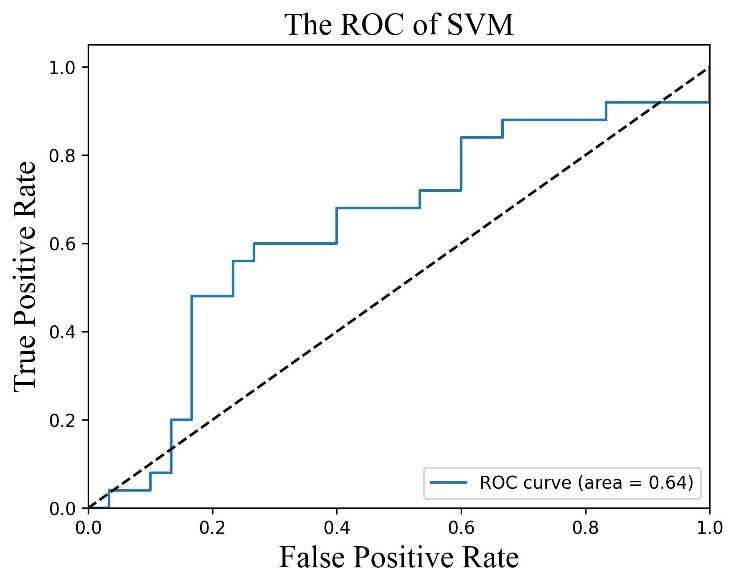


**Figure 2.** The ROC curve of the SVM models with only fNIRS features.
